# Multifaceted Primary Ciliary Dyskinesia—A Case Report

**DOI:** 10.3390/reports8010020

**Published:** 2025-02-09

**Authors:** Dinnar Yahya, Miroslava Benkova-Petrova, Aleksandar Petrov, Mari Hachmeriyan

**Affiliations:** 1 Department of Medical Genetics, Faculty of Medicine, Medical University of Varna, 9002 Varna, Bulgaria; mari.hachmeriyan@gmail.com; 2 Laboratory of Medical Genetics, UMHAT “Sv. Marina” Varna, Hristo Smirnenski Blvd 1, 9000 Varna, Bulgaria; 3 Second Department of Internal Diseases, Faculty of Medicine, Medical University of Varna, 9002 Varna, Bulgaria; benkova_miroslava@yahoo.com (M.B.-P.); alexander.petrov1@gmail.com (A.P.); 4 Clinic of Nephrology, University Multiprofile Hospital for Active Treatment “Sv. Marina”, Hristo Smirnenski Blvd 1, 9000 Varna, Bulgaria

**Keywords:** primary ciliopathy, whole-exome sequencing, genetic heterogeneity, genetic techniques, WES

## Abstract

**Background and Clinical Significance**: Ciliopathies are a heterogeneous group of diseases caused by damage to the primary cilium. Disorders of ciliary motility can lead to a wide range of clinical manifestations, including infertility, lateralization defects, lung infections, and more. Some ciliopathies associated with kidney disease include nephronophthisis, polycystic disease, and renal cell carcinoma. Since they are clinically and genetically diverse, their diagnosis may require a longer time and one or more genetic assays. **Case presentation**: We present the case of a 43-year-old man with a wide anamnesis, including unexplained nephrolithiasis, bronchiectasis, recurrent otitis media since infancy, appendicular lithiasis, and infertility. After a long history of various clinical examinations and consultations with diverse specialists, he was referred to genetic counseling. Whole exome sequencing (WES) revealed a homozygous pathogenic variant in the *RSPH3* gene—NM_031924.8:c.205-2A>G—which was later confirmed through Sanger sequencing. It is classified as pathogenic in widely used databases and is associated with primary ciliary dyskinesia. This condition can present nontypically, and the patients might suffer from an extensive diagnostic odyssey. Being mindful of its clinical and genetic heterogeneity can shorten the period until diagnosis. **Conclusions**: It is essential to have this condition included in differential diagnosis and involve specialists from the medical/clinical genetic department in a multidisciplinary team. Genetic confirmation through WES or another molecular genetic method is crucial for the therapeutic approach and to adequately perform genetic counseling for patients and their families.

## 1. Introduction

Primary ciliopathies are a group of genetically heterogeneous disorders caused by defects in the structure or function of primary cilia. The latter are microtubule-based organelles crucial in cellular signaling and developmental processes. Currently, pathogenic variants in at least 45 genes are known to be causative. Most commonly, they encode proteins involved in dynein complex structure—DNAH5 (15–29%), DNAI1 (2–10%), and *DNAH11* (6–9%) [1]. Numerous possible variants have been described, such as indels leading to a frameshift, nonsense truncating, or splice variants, eventually impairing the outer or inner dynein arm ultrastructure [2]. With a few exceptions, primary ciliopathies are usually inherited in an autosomal recessive manner [1]. These conditions are characterized by a wide range of clinical manifestations, reflecting the essential role of cilia in various tissues and organs. Common features of ciliopathies include renal cysts, retinal degeneration, polydactyly, liver fibrosis, and cerebellar malformations. The variability in clinical presentation often makes diagnosis challenging [3]. Thus, a multidisciplinary approach and thorough investigation of symptoms, laboratory, and other findings are essential for these patients.

The diagnosis of primary ciliopathies typically involves a combination of clinical evaluation, imaging studies, and genetic testing. Identifying the genetic mutations responsible for the condition is crucial, confirming the diagnosis, guiding prognosis, and providing genetic counseling. Depending on the clinical context and availability, genetic testing includes targeted panels, whole exome sequencing (WES), or whole genome sequencing [4]. WES, first described in 2009, is a widely used method that involves the sequencing of the protein-coding regions of the genome (exons). The human exome represents less than 2% of the genome, while containing approximately 85% of known disease-associated variants. These characteristics qualify the study as a cost-effective alternative to whole-genome sequencing. Thus, sequencing complete coding regions (exome) can reveal the diagnosis of many rare monogenic genetic diseases and predisposing variants of common and oncological diseases [5].

## 2. Case History

**Figure 1:**
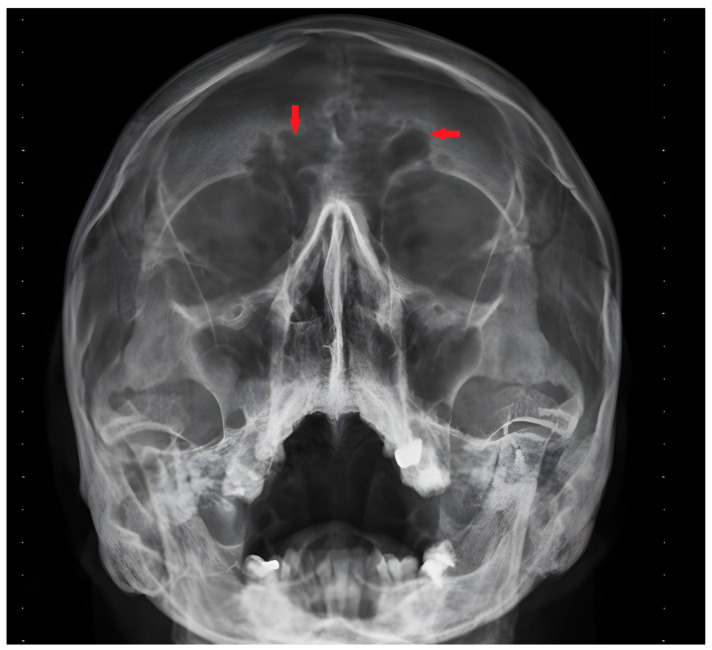
Frontal sinus infection—X-ray image. Red arrows indicate the sinuitis localization.

**Figure 2:**
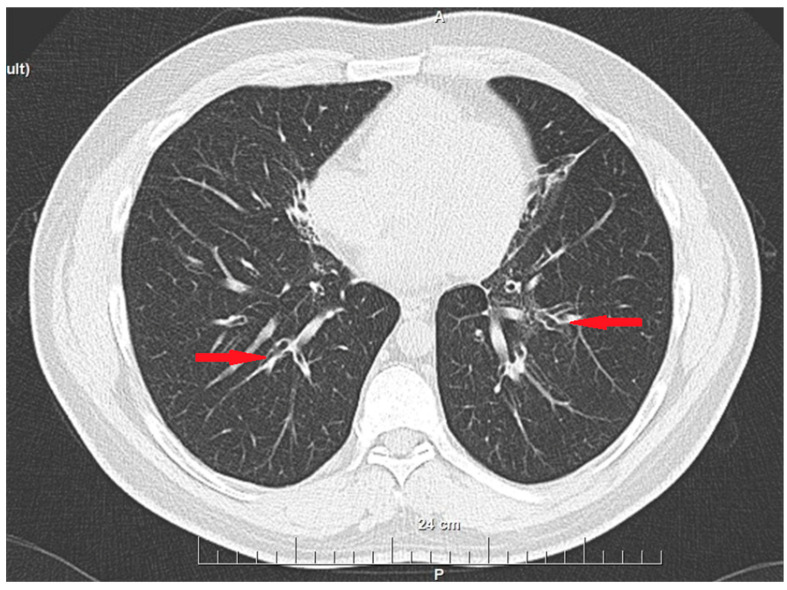
Bronchiectasis—CT scan image (axial plane). Red arrows indicate the dilated bronchi.

We present the case of a 43-year-old patient with multiple chronic complaints. Recurrent rhinosinusitis (Figure 1) and otitis media had been present since early childhood. He reported having an unexplained respiratory distress syndrome around birth and a persistent productive cough since infancy. He also had a history of bronchiectasis (Figure 2) and nephrolithiasis, as well as a stone with appendicular location. Additionally, the patient reported reduced hearing. Increased intraocular pressure and a residual thymus were also eventually found. Despite undergoing extensive medical management, including surgeries and long-term antibiotic therapies, the patient’s symptoms persisted, emphasizing the chronic and refractory nature of the disease. During his adulthood, he was diagnosed with infertility. His spermogram showed 99% non-motile spermatozoa and abnormal morphology in 99.5%. The consecutive testicular biopsy confirmed the asthenoteratozoospermia. Hypogonadotropic hypogonadism was also confirmed by laboratory testing. The couple opted for assisted reproduction through intracytoplasmic sperm injection, which was initially successful, but the embryo then showed an aneuploidy incompatible with life. Due to the heterogeneous clinical presentation and effect on multiple organs and systems, we considered primary ciliopathy and turned to genetic testing for a definitive diagnosis.

A medical geneticist consulted the patient to assess the case for a possible underlying genetic condition. Family history was negative, and pre-test genetic counseling was performed to explain the meaning of a probable genetic condition and its implications for the patient and his family. The scope and the limitations of the methods were also thoroughly communicated. Informed consent was then obtained from the index case. A molecular genetic test was ordered to confirm the clinical suspicion of a hereditary disease. We used DNA isolated from venous blood leukocytes via the salting out method and continued with outsourcing WES as a method of choice, given the genetic heterogeneity of the condition. Further data analysis with an in-house elaborate algorithm, including short-read alignment, mapping quality control, a copy number variations' pipeline, Ensembl’s variant effect predictor v106, and further variant calling and filtration of possible associated variants led to the discovery of a homozygous pathogenic variant in the *RSPH3* gene—NM_031924.8:c.205-2A>G. The variant is a transition in an AG acceptor splice site in intron 2, known to affect splicing, and is therefore deleterious. It has been reported in homozygous and compound heterozygous male patients with similar clinical manifestations [6]. Sanger sequencing was also performed to confirm the WES finding. Such variants in this gene in a homozygous or compound heterozygous state are associated with primary ciliary dyskinesia (PCD), OMIM #616481 [1,6]. PCD is a rare single-gene disorder with extreme locus heterogeneity. Clinically, it presents with early-onset recurrent respiratory infections, bronchiectasis, sinusitis, male infertility, and female subfertility. In males, the structure and motility of spermatozoa diminish the ability to fertilize an egg cell. Meanwhile, in the female reproductive system, ciliary dysfunction impairs oocyte and early transport, increasing the risk of ectopic pregnancies. In about half of the cases, lateralization defects are also observed—either heterotaxy or situs inversus [1]. The gene *RSP3* in Chlamydomonas is a protein kinase A-anchoring protein that binds ERK1/2 [7]. The described variant affects RNA splicing and impairs normal gene product synthesis. The protein normally participates in the highly conservative motile ciliary structures—central microtubules and radial spokes [6]. Their presence in the respiratory, reproductive, cardiovascular, and central nervous systems, as well as the thymus, explains a single-gene condition’s so-called pleiotropic effect—a multisystem phenotype [8]. In this case, the gene on chromosome 6 has both copies functionally affected—an autosomal recessive type of inheritance. The origin of these two identical variants in the studied patient is probably related to the obligate heterozygosity of his parents or a combination of an inherited and a de novo variant. Despite our wish to clarify the origin, the patient declined, since it was related to a further financial burden to this initially patient-paid testing. Additionally, since he was of reproductive age and had already undergone one assisted reproduction procedure with his wife, the risks of re-occurrence needed clarification. Potential success in the next procedure would lead to an obligate carrier child in every single pregnancy, since the child would necessarily inherit one pathogenic variant from the affected homozygous father. The risk of having an affected child is low and depends on the population frequency of this or another pathogenic variant in the *RSPH3* gene. The results and risks mentioned above were explained via post-test genetic counseling. After the patient’s consent, they were also discussed with the referring specialist to plan his diagnosis-based management.

## 3. Discussion

Primary ciliopathies are a group of inherited disorders caused by defects in the structure or function of primary cilia, leading to a wide range of clinical manifestations across multiple organ systems. Due to the genetic heterogeneity and phenotypic overlap of these conditions, accurate diagnosis can be challenging. The presented case was mainly difficult due to the initial lack of information about the entire complaint spectrum. In our experience, patients tend to focus on the symptoms related to the specialty of the physician they are meeting. For example, kidney stones were the main reason for our patients meeting a nephrologist. Still, considering his recurrent respiratory and ear infections, and later found infertility, were crucial for solving the “puzzle”. The lack of laterality defects could also be confusing, as this is only seen in half of the patients [1]. Also, this group of conditions is characterized by a wide differential diagnosis. The neonatal respiratory distress syndrome, bronchiectasis, productive cough, and infertility in our patient could raise suspicion for cystic fibrosis. On the other hand, the reduced thymus and frequent respiratory infections could bring forward the possibility of immunodeficiency syndrome. Some could also suspect a combination of multiple nosological entities to explain the entire clinical plethora. Gathering all this information demanded both time and good multidisciplinary collaboration. The case we observed represents the need to actively investigate the full plethora of signs and symptoms and personal and family histories before building the differential diagnosis and diagnostic approach.

The next challenging step when suspecting PCD is choosing the genetic confirmation method. Given the number of genes possibly affected, the test must have great coverage. The best would be whole-genome sequencing, WES, or targeted panel testing. Still, these methods are imperfect, and their scope and limitations must be understood well by physicians and consulted patients.

Another important factor is the financing of this analysis. In Bulgaria, most of the genetic analyses are patient-paid. The only exceptions are karyotyping and a limited number of targeted molecular tests for inpatient pediatric patients and adult ones with hematological malignancies. Due to these unfavorable economic circumstances, patients must understand why such a costly method is required to confirm the suspected condition, even more so than if covered by national health care. In our opinion, one of the superior approaches, considering the value–price ratio, is WES. This method has emerged as a powerful diagnostic tool for primary ciliopathies. WES focuses on sequencing the protein-coding regions of the genome, which are most likely to harbor pathogenic variants. It is precious for ciliopathies because it allows for the simultaneous examination of many genes associated with these disorders, reducing the time and cost compared to traditional methods, namely PCR and Sanger sequencing, which target specific genes individually [9,10]. Still, Sanger sequencing can be a gold standard for confirming WES findings.

WES application in clinical settings has significantly improved the diagnostic yield for primary ciliopathies, particularly in cases where traditional diagnostic approaches fail to identify a clear genetic cause. While targeted gene panel testing could be an excellent and fully sufficient choice for genetic testing in cases of clear clinical suspicion and known causative genes, WES has been instrumental in identifying novel mutations and expanding the known genetic landscape of these conditions. According to published studies, WES can detect causative mutations in most patients with suspected ciliopathies, leading to more precise diagnoses and better-informed clinical management [11,12,13].

Moreover, WES demonstrated a high yield in identifying cases with atypical presentations or overlapping features with other genetic disorders, further underscoring its importance in the diagnostic process. This method’s integration into routine clinical practice for suspected ciliopathies allows for a comprehensive genetic evaluation, which is crucial for guiding treatment decisions, family planning, and understanding the prognosis of these complex disorders [4].

## 4. Conclusions

WES has proven to be a critical diagnostic tool for primary ciliopathies, significantly advancing our understanding and management of these complex, genetically heterogeneous disorders. Its ability to uncover novel causative variants and broaden the genetic landscape of these conditions enhances diagnostic accuracy. It provides valuable insights into disease mechanisms, guiding more personalized and effective treatment strategies. As such, WES stands as a cornerstone in the diagnostic process for primary ciliopathies, offering hope for a shorter diagnostic odyssey and earlier and more precise interventions. The non-classical presentation of the disease in our patient was a great example of the much-needed critical thinking extended differential diagnosis and multidisciplinary collaboration.

## Data Availability

The data presented in this study are available on request from the corresponding author due to ethical considerations related to the involved subject.

## Abbreviations

PCD	Primary ciliary dyskinesia
WES	Whole exome sequencing
